# Lipoprotein(a) Concentration and Achieving Target Values of Low-Density Lipoprotein Cholesterol Calculated by Different Equations

**DOI:** 10.3390/diseases14020041

**Published:** 2026-01-27

**Authors:** Olga I. Afanasieva, Alexandra V. Tyurina, Elena A. Klesareva, Marat V. Ezhov, Sergei N. Pokrovsky

**Affiliations:** 1 Institute of Experimental Cardiology, National Medical Research Center of Cardiology n.a. acad. E.I. Chazov, Ministry of Health of the Russian Federation, Moscow 121552, Russia; hea@mail.ru (E.A.K.); dr.pokrovsky@mail.ru (S.N.P.); 2 A.L. Myasnikov Institute of Clinical Cardiology, National Medical Research Center of Cardiology n.a. acad. E.I. Chazov, Ministry of Health of the Russian Federation, Moscow 121552, Russia; alex.tyurina.cardio@yandex.ru

**Keywords:** lipoprotein(a), LDL-cholesterol, Martin–Hopkins equation, Sampson equation, Friedewald formula, reclassification

## Abstract

Background: Low-density lipoprotein cholesterol (LDL-C) is a major cardiovascular risk factor and an indicator of hypolipidemic therapy effectiveness. However, direct and calculated methods for determining “LDL-C” present the sum of the cholesterol in all apoB-containing lipoproteins, including lipoprotein(a) [Lp(a)]. There has been an ongoing debate about the correctness of LDL-C in patients with elevated Lp(a) concentrations up to now. The aim of this study was to evaluate the effect of Lp(a) concentration on the LDL-C calculated by different equations. Methods: The study included the results of fasting lipids and Lp(a) concentration of 566 measurements from 283 patients (before and after lipid-lowering therapy prescribing, after exclusion of 17 patients with incomplete data). LDL-C and LDL-C corrected for Lp(a)-cholesterol (LDL-C_corr_) were calculated by Friedewald, Martin–Hopkins, and Sampson equations. Results: We assessed 566 measurements of lipids and Lp(a). The number of values reclassified to a higher risk category was 10% and 13% with Martin–Hopkins and Sampson equations compared to the Friedewald formula. The percentage of Lp(a)-cholesterol (Lp(a)-C) in the LDL-C calculated by three formulas was up to 90% or more depending on the concentration of LDL-C and Lp(a). When stratified by clinically significant LDL-C thresholds, the proportion of values LDL-C_corr_ reclassified to a lower risk category ranged from 30 to 59%. Conclusion: Comparison of LDL-C concentrations calculated by Friedewald, Martin–Hopkins, and Sampson equations showed high consistency in patients without elevated triglycerides. The LDLcorr is reasonable to use in patients with Lp(a) concentration ≥ 30 and ≥41 mg/dL when using the Martin–Hopkins and Sampson equations, respectively. These data may help clinicians interpret LDL-C goal attainment in patients with elevated Lp(a) and avoid misclassification driven by the Lp(a)-cholesterol component.

## 1. Introduction

Low-density lipoprotein cholesterol (LDL-C) is a major determinant of atherosclerotic cardiovascular disease risk and the primary treatment target in lipid-lowering therapy. In everyday practice, decisions about therapy initiation and intensification are frequently driven by whether a patient is above or below guideline-defined LDL-C thresholds; therefore, even moderate systematic error in LDL-C estimation may translate into clinically relevant misclassification of risk category and treatment intensity. Most clinical laboratories still report LDL-C as a calculated value, most commonly using the Friedewald equation [1,2,3]. However, the Friedewald approach assumes a fixed ratio of triglyceride (TG) to very low density lipoprotein cholesterol (VLDL-C) and becomes less accurate with increasing TG levels and in settings where LDL-C is low, which is particularly important in the era of intensive LDL-lowering therapy [4].

Several improved equations have been developed to reduce calculation error, including the Martin–Hopkins method with an adjustable TG:VLDL-C factor and the Sampson (NIH) equation, which were shown to improve LDL-C estimation across relevant lipid phenotypes compared with Friedewald [5,6,7].

However, all direct and calculated methods for determining “LDL-C” represent the sum of the cholesterol contained in three lipoproteins: LDL, intermediate-density lipoproteins (IDL), and lipoprotein(a) [Lp(a)]. And if in a fasting blood sample for patients without a diagnosis of familial dysbetalipoproteinemia, IDL-C usually makes a very limited contribution and can be neglected, this is not the case for Lp(a).

Elevated lipoprotein(a) [Lp(a)] represents a special clinical scenario because cholesterol carried by Lp(a) contributes to the reported “LDL-C” (whether measured directly or calculated), whereas the Lp(a) particle itself is largely genetically determined and responds poorly to standard LDL-lowering strategies. As a result, clinicians may face an apparent paradox: a patient receiving intensive lipid-lowering therapy has “LDL-C” above target, yet further escalation of LDL-directed therapy may provide diminishing returns if a substantial fraction of the reported LDL-C is attributable to Lp(a)-cholesterol rather than LDL particles. This may affect clinical interpretation of residual risk, the perceived effectiveness of therapy, and decisions about additional testing and treatment escalation, especially in very high risk patients managed to stringent LDL-C targets.

Since the discovery of Lp(a) and up to the present time, there has been an ongoing debate about the correctness of using LDL-C in patients with elevated Lp(a) concentrations. Lipoprotein(a) is a complex particle in which apolipoprotein B100, which is part of the LDL-like particle, is linked by a single disulfide bond to unique apolipoprotein(a).

Lp(a), as well as LDL, contains proteins, cholesterol and its esters, phospholipids, and triglycerides, while cholesterol and its esters make up about a third of the total mass of Lp(a) [8]. LDL-C obtained by calculation, or measured by direct method, is the sum of LDL-C and Lp(a)-cholesterol (Lp(a)-C) [9,10]. When the concentration of Lp(a) is low, the obtained values of LDL-C are correct. On the contrary, when Lp(a) level is elevated, the error in the determination of LDL-C can be quite significant [11,12]. The contribution of Lp(a)-C to the LDL-C is most pronounced in patients with high Lp(a) concentrations who are on aggressive hypolipidemic therapy, which dramatically reduces plasma LDL-C content, but has no effect on Lp(a) level.

Thus, the issue of achieving the “target level” of LDL-C in patients with elevated Lp(a) concentration becomes extremely relevant, but there has been an ongoing debate about the correctness of determination of LDL-C in these patients up to now. From a clinical perspective, this may lead to misclassification of goal attainment and potentially inappropriate treatment decisions: LDL-C can appear above guideline-recommended thresholds because it includes the cholesterol carried by Lp(a), particularly in patients receiving intensive lipid-lowering therapy where LDL particles are reduced but Lp(a) remains unchanged. Therefore, separating the LDL-particle cholesterol from the Lp(a)-cholesterol component could improve the clinical interpretation of whether a patient has truly achieved the LDL-C target and may help clinicians better judge residual risk attributable to Lp(a). We hypothesized that (i) Lp(a) correction would systematically shift patients to lower LDL-C categories (“down-classification”), and (ii) the magnitude of reclassification would differ across commonly used LDL-C equations (Friedewald, Martin–Hopkins, and Sampson), particularly in strata with elevated Lp(a). Accordingly, this study aimed to quantify how Lp(a) affects LDL-C calculated by different equations and to estimate the extent of clinically relevant reclassification across LDL-C thresholds.

The aim of this study was to evaluate the effect of Lp(a) concentration on the LDL-C calculated by different equations.

## 2. Materials and Methods

This study was a secondary analysis of fasting lipid profile and lipoprotein(a) [Lp(a)] measurements obtained from 300 patients examined at two time points: before and after initiation of lipid-lowering therapy. Early manifestation of coronary heart disease (CHD) was documented in 200 patients, whereas 100 patients without CHD had no hemodynamically significant stenosis of the coronary and peripheral arteries. The baseline characteristics of the examined patients are presented in Table 1. The study was conducted in the National Medical Research Center of Cardiology (Moscow, Russia).

**Figure 1:**
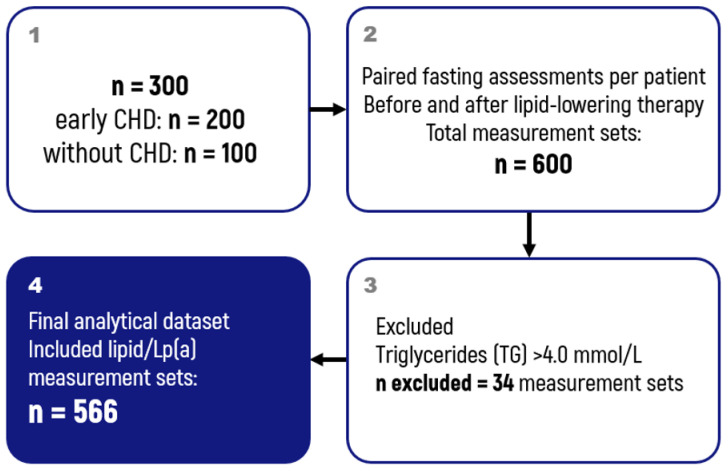
Study design.

Inclusion criteria were (i) availability of paired measurements at two time points (before and after initiation of lipid-lowering therapy) for the same patient; and (ii) availability of fasting lipid profile and Lp(a) results. Because the Friedewald equation is not recommended at high triglyceride (TG) concentrations, measurements with TG > 4.0 mmol/L were excluded from the final analytical dataset. As a result, 566 lipid measurements were included in the final analysis (Figure 1).

The concentrations of lipid spectrum parameters, namely total cholesterol (TC), TG, and high-density lipoprotein cholesterol (HDL-C) in the blood serum, were measured by the enzymatic colorimetric method on the Architect C-8000 (Abbott, Chicago, IL, USA) biochemical analyzer.

Non-HDL-C was calculated according to the following formula:

Non-HDL-C = TC − HDL-C, mmol/L

LDL-C level was calculated using the following formulas:

Friedewald formula:

LDL-C = TC − HDL-C − TG/2.2

Martin–Hopkins formula:

LDL-C = TC − HDL-C − TG/f

where f is the adjusted value from 3.1 to 11.9 (result in mg/dL) [13].

Sampson formula:

LDL-C = TC/0.948 − HDL-C/0.971 − (TG/8.56 + TG × non-HDL-C/2140 − TG^2^/16,100) − 9.44, mmol/L

In addition, we calculated the level of corrected LDL cholesterol (LDL-C_corr_), taking into account the Lp(a)-cholesterol [14]:

LDL-C_corr_ = LDL-C − Lp(a) × 0.3, mmol/L

where Lp(a) is the concentration of Lp(a) in mg/dL [15].

Lp(a) concentration was measured by enzyme immunoassay with monospecific polyclonal sheep antibodies against human Lp(a) [16] with modifications. For this purpose, the plate (Sovtech, Moscow, Russia) coated with antibodies against human Lp(a) was washed with 0.15 M NaCl and incubated with 300 µL of phosphate buffer containing bovine serum albumin and Tween-20 for 1 h at 37 °C. The tested serum samples and a control sample with known Lp(a) concentration were diluted 300 times and incubated for 30 min at 37 °C. After washing, the plate was incubated for 30 min with anti-Lp(a) antibodies conjugated with peroxidase. For detection, the plate was incubated for 20 min, without shaking, at 18–25 °C, with single-component TMB solution (Niopik, Moscow, Russia). The reaction was stopped by adding 100 μL of 5% sulfuric acid solution, and the optical density was recorded on a plate spectrophotometer (Multiskan Go, Thermo Scientific, Waltham, MA, USA) at a wavelength of 450 nm. The method was previously validated against reference materials TruLab Lp(a) Levels 1 and 2 (DiaSys, Holzheim, Germany) and parallel measurement of 150 serum samples from volunteers on biochemical analyzer Architect-C 8000 (Abbott, Chicago, IL, USA).

Statistical data processing was performed using the MedCalc 23.2.6 package (MedCalc Software Ltd., Oostende, Belgium). Lin’s concordance correlation coefficient (CCC) was performed to test the relationship between LDL-C values calculated using different equations, since the values were continuous, and their distribution was close to normal. To determine the threshold concentrations of Lp(a) that affect the reclassification of LDL-C_corr_, the analysis of the receiver operating characteristic curves (ROC analysis) was used. Cohen’s kappa coefficient was calculated to analyze and construct consistency tables between the LDL-C and LDL-C_corr_ categories. Due to potential bias in Cohen’s kappa when category distributions are unbalanced, Gwet’s AC1 coefficient was additionally calculated. For regression analysis of uncorrected and LDL-C corrected for Lp(a)-cholesterol least-product regression (LPR; also known as geometric mean regression or reduced major axis regression) was used instead of ordinary least squares (OLS) regression. LPR is appropriate when both variables (LDL-C and LDL-C_corr_) are measured with error, as both are calculated quantities. The slope of LPR is calculated as m = SD(y)/SD(x), and the y-intercept as intercept = MEAN(y) − m × MEAN(x). This approach is consistent with the method used for calculating Lin’s concordance correlation coefficient.

## 3. Results

Analysis of TG levels in the overall cohort identified 17 patients with levels greater than 4 mmol/L before and after prescription of hypolipidemic therapy, respectively. Given the limitations of using the Friedewald equation for elevated TG values, the results of 566 lipid measurements were included in the final study. To assess the agreement between the three calculation methods, LDL-C values were divided into eight categories according to clinically significant cutoff values (Table 2).

**Figure 2:**
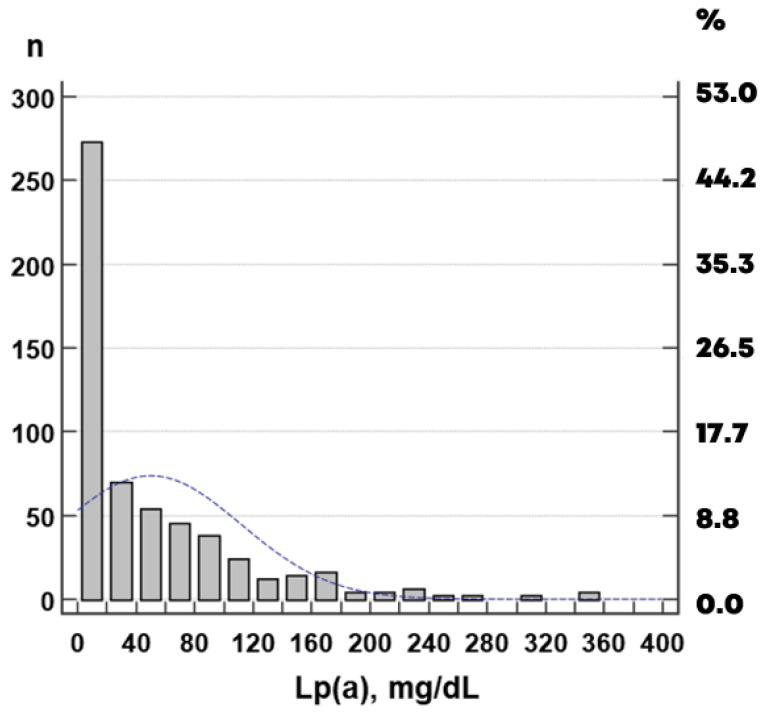
Distribution of Lp(a) concentration. The blue dotted line shows the Gaussian distribution.

The number of values reclassified to a higher clinical category was 13% when using the Martin–Hopkins formula for calculation and 10% for the Sampson formula. The agreement between LDL-C categories calculated by different formulas was quite high, with Cohen’s kappa coefficients of 0.78, 0.84, and 0.90 when comparing the Martin–Hopkins and Friedewald, Sampson and Friedewald, and Sampson and Martin–Hopkins equations, respectively. Plasma Lp(a) concentration varies over a very wide range, which, for our sample, ranged from 1 to 354 mg/dL, with a median (interquartile range) of 24 (8–74) mg/dL (Figure 2).

**Figure 3:**
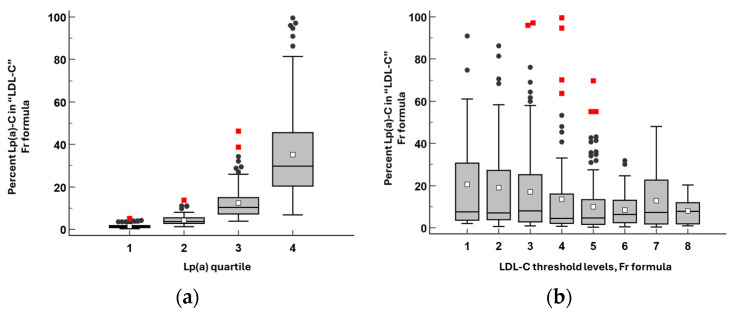

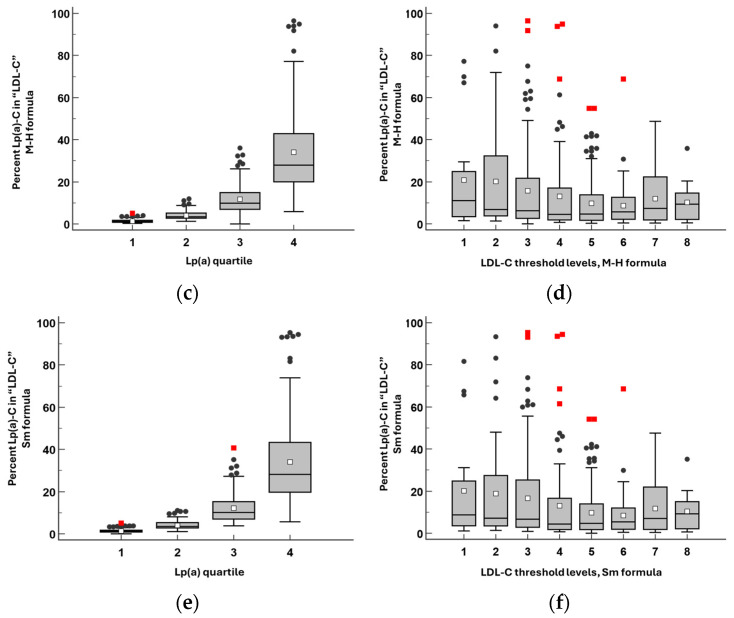
The percentage of lipoprotein(a)-cholesterol [Lp(a)-C] in LDL-C calculated by the Friedewald (**a**,**b**), Martin–Hopkins (**c**,**d**), and Sampson (**e**,**f**) formulas as a function of Lp(a) quartiles (**a**,**c**,**e**) and clinically relevant LDL-C threshold categories (**b**,**d**,**f**; thresholds as in Table 1). Data are presented as box-and-whisker plots in which the solid horizontal line within each box represents the median and the white dot represents the mean; the lower and upper edges of the box represent the 25th and 75th percentiles (interquartile range, IQR), respectively. Whiskers extend to the smallest and largest values within 1.5 × IQR from the box edges. Values further than 1.5 × IQR are shown as outliers (black circles), whereas values further than 3 × IQR are shown as gross outliers (red squares).

Depending on the concentration of LDL-C and Lp(a), the contribution of Lp(a)-C to the LDL-C indicator calculated using three formulas can reach 90% or more (Figure 3).

When stratified by clinically significant LDL-C thresholds calculated using different equations and considering Lp(a)-C, the proportion of values reclassified to a lower LDL-C category ranged from 30 to 59% (Table 3).

**Figure 4:**
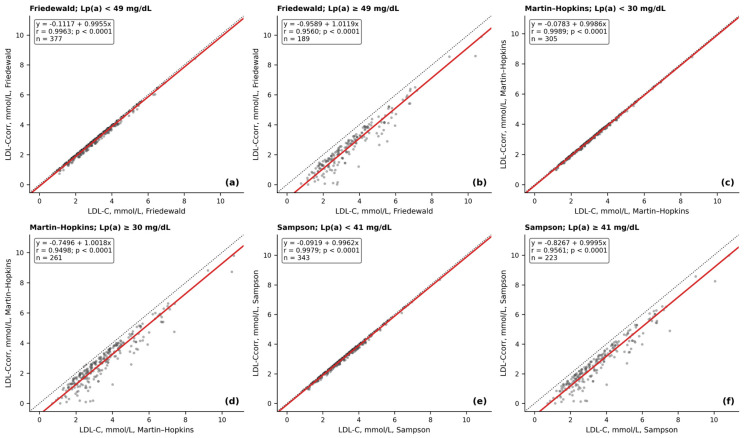
Least-product (reduced major axis, RMA) regression plots comparing uncorrected LDL-C (*x*-axis) and LDL-C corrected for Lp(a)-cholesterol (LDL-C_corr_; *y*-axis), calculated using the Friedewald (**a**,**b**), Martin–Hopkins (**c**,**d**), and Sampson (**e**,**f**) equations. Panels show results stratified by the formula-specific Lp(a) cutoff values derived from ROC analysis: Friedewald, (**a**) Lp(a) < 49 mg/dL and (**b**) Lp(a) ≥ 49 mg/dL; Martin–Hopkins, (**c**) Lp(a) < 30 mg/dL and (**d**) Lp(a) ≥ 30 mg/dL; and Sampson, (**e**) Lp(a) < 41 mg/dL and (**f**) Lp(a) ≥ 41 mg/dL. Each point represents one lipid measurement. The red line indicates the LPR/RMA fit (slope m = SD(y)/SD(x); intercept b = mean(y) − m × mean(x)).

Based on the results of ROC analysis for each of the three formulas, Lp(a) concentrations were found with the maximum sensitivity and specificity associated with the reclassification of the LDL-C value to lower threshold levels. The concentration of Lp(a) ≥ 49 mg/dL allowed us, with a sensitivity of 65% and a specificity of 86%, to reclassify the LDL-C level calculated using the Friedewald equation to a lower one. There was no systematic error in the calculation of LDL-C in the samples with Lp(a) level < 49 mg/dL, whereas the systematic error was 0.81 mmol/L in the samples with Lp(a) concentration ≥ 49 mg/dL (Figure 4a,b).

For calculation of LDL-C using the Martin–Hopkins formula, Lp(a) concentration ≥ 30 mg/dL with a sensitivity of 79% and specificity of 73% allowed for the reclassification of LDL-C levels. The systematic error in this case was 0.65 mmol/L and was absent in the sample with Lp(a) levels < 30 mmol/L (Figure 4c,d).

Using the Sampson formula, the threshold level of Lp(a) allowing for reclassification of LDL-C to a lower level with a sensitivity of 77% and a specificity of 80% was 41 mg/dL. As in previous cases, the systematic error in calculating LDL-C was 0.71 mmol/L in the sample with Lp(a) concentration ≥ 41 mg/dL (Figure 4e,f).

Lin’s concordance correlation coefficient (CCC) between LDL-C and LDL-C_corr_ showed moderate agreement for all three formulas: Friedewald (CCC = 0.904; 95% CI, 0.887–0.918), Martin–Hopkins (CCC = 0.908; 95% CI, 0.892–0.921), and Sampson (CCC = 0.910; 95% CI, 0.894–0.923). The moderate agreement (rather than almost-perfect agreement) reflects the systematic difference introduced by Lp(a) correction, with mean reductions of approximately 0.39 mmol/L (SD: 0.47 mmol/L). Cross-formula comparisons showed substantial-to-almost-perfect agreement, both before and after Lp(a) correction. Martin–Hopkins and Sampson formulas demonstrated almost perfect agreement (CCC = 0.998 for LDL-C; CCC = 0.997 for LDL-C_corr_), while Friedewald showed substantial agreement with both formulas (CCC > 0.97).

**Figure 5:**
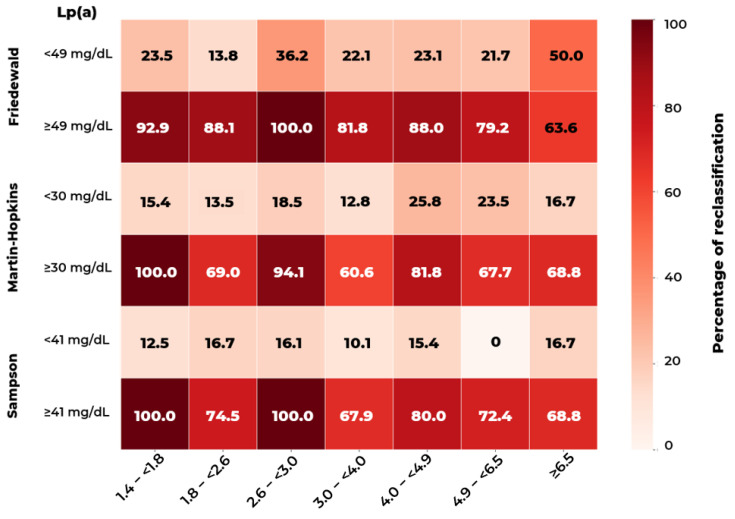
Percentage of outcomes reclassified to lower LDL cholesterol when using the Lp(a) cholesterol adjustment (“LDL_corr_ cholesterol”).

Thus, relative to the identified threshold values of Lp(a) concentrations in the calculation of LDL-C, LDL_corr_ could be correctly reclassified to lower ranges of 61 to 100% of values (Figure 5).

**Figure 6:**
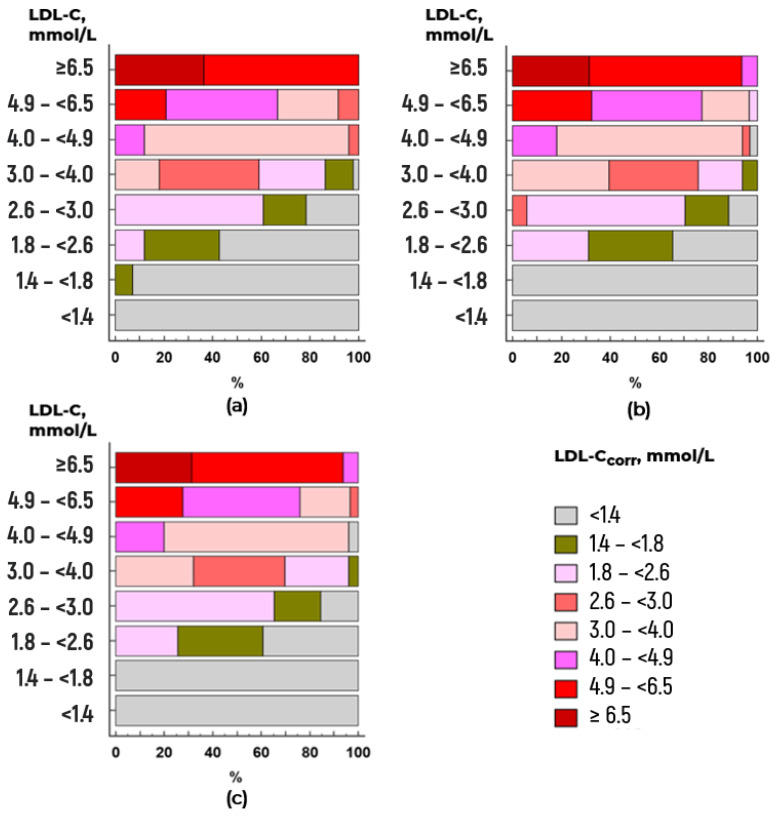
Correspondence of clinically relevant threshold values of LDL-C and LDL-C_corr_ calculated by (**a**) the Friedewald formula at Lp(a) concentrations ≥ 49 mg/dL, Cohen’s kappa coefficient of 0.04 (95% CI −0.02 to 0.11); (**b**) the Martin Hopkins formula for Lp(a) concentrations ≥ 30 mg/dL, Cohen’s kappa coefficient of 0.15 (95% CI 0.09 to 0.21); and (**c**) Sampson’s formula for Lp(a) concentrations ≥ 41 mg/dL, Cohen’s kappa coefficient of 0.11 (95% CI 0.05 to 0.18).

The consistency between the clinically relevant LDL-C and LDL-C_corr_ categories was high to very high (Cohen’s kappa coefficients in the range of 0.81–1.00) at Lp(a) concentrations below the calculated cutoff values and completely disappeared (coefficients < 0.2) at Lp(a) concentrations above the cutoff values (Figure 6a–c; Tables S1–S3).

Table 4 summarizes the agreement between clinically relevant LDL-C categories derived from the original and Lp(a)-corrected LDL-C estimates. When exact category matching was required, unweighted Cohen’s kappa was low (κ = 0.046 for Friedewald at Lp(a) ≥ 49 mg/dL; κ = 0.161 for Martin–Hopkins at Lp(a) ≥ 30 mg/dL; κ = 0.121 for Sampson at Lp(a) ≥ 41 mg/dL), with observed agreement of 0.169, 0.284, and 0.242, respectively. In contrast, quadratic-weighted kappa was high (0.753–0.796), indicating that most discordances represented shifts to adjacent LDL-C categories rather than large cross-category changes. To address the known sensitivity of κ to marginal category distributions, we additionally computed Gwet’s AC1; AC1 values (0.054–0.185) were consistent with the unweighted κ results (Supplementary Figure S1).

## 4. Discussion

Currently, the Friedewald formula is widely used in clinical practice to calculate LDL-C, despite its limitations [4]. The recommendations of the European Atherosclerosis Society and the European Federation of Clinical Chemistry and Laboratory Medicine suggest using the Martin–Hopkins formula to calculate LDL-C, primarily in patients with LDL-C levels < 1.8 mmol/L and/or TG levels in the range of 2.0 to 4.5 mmol/L [15]. Our study demonstrated minimal differences when comparing the results obtained using the Friedewald formula, as well as the Martin–Hopkins and Sampson formulas in the range of LDL-C concentrations from 0.8 to 10.7 mmol/L and TG from 0.5 to 3.9 mmol/L. Such high agreement (over 95%) between the three formulas can be explained by the fact that we excluded patients with triglyceride levels ≥ 4.0 mmol/L from the study.

Similar results were obtained in a study with 10,000 participants (70.6% men), where the agreement between LDL-C levels was lowest when comparing the Martin–Hopkins and Friedewald equations (88.4%), higher when comparing the Friedewald and Sampson formulas (92.3%), and highest agreement when comparing the Martin–Hopkins and Sampson equations (95.1%). Compared with the Friedewald equation, the Martin–Hopkins and Sampson formulas showed a high proportion of upward reclassification (10.8% and 7.5%, respectively) [17].

In a study involving 5,051,467 participants and comparing 23 equations for calculating LDL-C levels, the Martin–Hopkins equation was the most accurate in determining LDL-C levels for classification into a risk category (89.6%), followed by the Sampson (86.3%), Chen (84.4%), Poivilai (84.1%), DeLong (83.3%), and Friedewald (83.2%) equations [18]. Taken together, these data support the practical concept that, even in populations with acceptable overall agreement, the choice of LDL-C equation may shift individual patients across clinically relevant decision thresholds, especially at lower LDL-C ranges, where treatment targets are strict.

In the era of innovative genetically engineered drugs, the correct answer to the question, “what is the real level of LDL-C in my patient?” is more relevant than ever in the framework of a personalized approach to cardiovascular risk assessment. Often against the background of maximal hypolipidemic therapy, it is not possible to achieve the target level of LDL-C, because a significant proportion of “LDL-C” is represented by Lp(a)-C, the concentration of which is resistant to modern hypolipidemic agents.

From the patient’s perspective, the clinical impact of our findings is straightforward: the reported laboratory value “LDL-C” may partially represent Lp(a)-cholesterol, and therefore, it may not fully reflect the number of LDL particles that is responsive to standard LDL-lowering therapy. In individuals with elevated Lp(a), the same measured LDL-C can correspond to different “true” LDL-C burdens, which may lead to apparent “failure” to reach LDL-C targets despite high adherence and maximally tolerated therapy. This issue becomes particularly important in very high risk patients managed to stringent LDL-C goals, where intensification decisions (e.g., additional non-statin therapy, frequency of monitoring, and assessment of residual risk) are often triggered by crossing a narrow numerical threshold. Our data demonstrate that Lp(a)-C can account for a substantial fraction of the measured/calculated LDL-C (up to ≥90% in certain combinations of LDL-C and Lp(a)), and that LDL-C correction may move 30–59% of results to a lower LDL-C category when evaluated against clinically used thresholds. Therefore, measuring Lp(a) and considering the potential contribution of Lp(a)-C can help clinicians communicate more accurately with patients about why LDL-C targets may appear unattainable and how to individualize next therapeutic steps.

According to expert consensus documents, recalculation of LDL-C to account for potential Lp(a)-C contribution may be considered in selected clinical scenarios, such as suspected high Lp(a) concentration or unexpectedly poor apparent LDL-C response to lipid-lowering therapy [15]. The main limitation of said formulation is the assumption that cholesterol constitutes approximately 30% of the mass of the Lp(a) particle [8,9,19,20,21]. The results of a study using a direct method of measuring the concentration of Lp(a)-C using monoclonal antibodies against apo(a) showed that the proportion of cholesterol in the Lp(a) composition can vary over a wide range, from 6 to 57% [10]. The conceptual framework that “LDL-C” represents LDL-C plus Lp(a)-C is increasingly emphasized in the context of contemporary intensive lipid-lowering therapy, where achieved LDL-C values may be very low and the relative contribution of Lp(a)-C to the reported LDL-C becomes proportionally larger. Based on these limitations, the European Atherosclerosis Society does not recommend routine use of the Dahlen formula to correct LDL-C concentration. Exceptions are patients with clinical signs of familial hypercholesterolemia (FH) and elevated Lp(a) levels, as well as subjects with baseline high Lp(a) levels and resistance to statins [22].

The use of LDL-C correction in patients with suspected FH has been shown in several studies. In a study including 281 patients with FH, correction of LDL-C concentration with consideration of Lp(a)-C increased the diagnostic accuracy of the Dutch Lipid Clinics and Simon Broome criteria [23]. Among 206 patients from the Russian SHCC registry, 13 (6%) patients had their diagnosis changed to unlikely, and 22 of 65 (34%) patients with Lp(a) concentrations ≥ 40 mg/dL had their diagnosis reclassified with a decreased probability of true SHCC [24]. Correction of LDL-C to clarify or exclude the diagnosis of FH may also avoid expensive genetic sequencing. Additional evidence from laboratory and clinical datasets suggests that measurable Lp(a)-C can shift patients into lower LDL-C-based diagnostic categories, potentially reducing false-positive phenotypic classification and unnecessary cascade testing in families.

In our study, we calculated, for the first time, the Lp(a) threshold levels that influence the reclassification of LDL-C_corr_ level according to different target values for each of the three equations used to calculate LDL-C. Understanding the threshold level at which Lp(a) concentration can influence LDL-C is important. For example, the recent study [25] evaluated the effect of Lp(a), LDL-C, and LDL-C_corr_ on the risk of CHD in a pooled European cohort (*n* = 68,748). Based on the results of the study, the authors concluded that the correction of LDL-C levels considering Lp(a)-C does not provide additional information for assessing the risk of coronary heart disease at the population level. It should be noted that the median (interquartile range) of Lp(a) concentration in this cohort was 9.3 (4.2–20.4) mg/dL, while the 90th percentile was 43.5 mg/dL. This means that only in 10% of the sample could Lp(a)-C correct LDL-C by a value of >0.3 mmol/L with a median LDL-C value of 3.5 (2.9–4.2) mmol/L. In the above study, the significant association between CHD and LDL-C_corr_ in the range of 2.72–3.26 mmol/L lost significance (*p* = 0.07). The relative risk was 1.30 (1.08–1.55) *p* = 0.0045 for LDL-C and 1.18 (0.99–1.41) *p* = 0.068 for LDL-C_corr_ [25]. These data underline that LDL-C correction may not meaningfully improve coronary risk prediction at the population level, particularly when Lp(a) concentrations are predominantly low; however, they do not negate potential utility in selected high-Lp(a) clinical subgroups in whom Lp(a)-C constitutes a materially relevant fraction of reported LDL-C. In a large meta-analysis involving 18,043 patients, including 5390 individuals with cardiovascular disease, based on the results of five landmark statin trials—CARDS (Collaborative Atorvastatin Diabetes Study), 4D (German Diabetes and Dialysis Study), LIPID (Long-Term Intervention with Pravastatin in Ischemic Disease) MIRACL (Reduction of Myocardial Ischemia with Aggressive Cholesterol Lowering) study, and 4S (Scandinavian Simvastatin Survival Study)—it was shown that the level of LDL-C_corr_ adjusted for Lp(a)-C content is not a prognostic factor for cardiovascular diseases, in contrast to “LDL-C” [26].

In our study, which included patients with early manifestation of CHD, 45% of the sample had an elevated Lp(a) concentration > 30 mg/dL. Moreover, the contribution of Lp(a)-C to LDL-C, and hence the error in the determination of LDL-C concentration and the possibility of reclassification, was particularly pronounced higher in the range, up to 2.6 mmol/L. This is especially important for high-risk patients, because for them, intensification of standard lipid-lowering therapy will not lead to achievement of target LDL-C levels. In the largest study of the prevalence of elevated Lp(a) concentrations in the USA (*n* = 531,144, age 57 [46; 67] years, 48% men), LDL-C was measured directly and calculated with the Dalen correction LDL-C_corr_. The LDL-C_corr_ level in the corresponding Lp(a) quintiles (Q1 < 6, 6 ≤ Q2 < 12, 12 ≤ Q3 < 24, 24 ≤ Q4 < 60, Q5 ≥ 60 mg/dL) was 1.0%, 3.5%, 10.5%, 11.2%, and 27.7% lower than the laboratory-determined LDL-C level [27].

Thus, the need for accurate LDL-C determination, especially in patients with elevated Lp(a) concentrations, remains an important clinical science challenge to optimize therapy in the era of innovative targeting drugs. An accurate value of LDL-C without the contribution of Lp(a)-C is also necessary to reduce global cardiovascular risk and age-adjust for initiation of hypolipidemic therapy in patients with elevated Lp(a) concentrations [22]. The ability of statins to increase Lp(a) concentrations also necessitates estimation of the true value of LDL-C, especially when patients fail to achieve target levels despite high adherence to treatment. This phenomenon has been demonstrated in meta-analyses of randomized statin trials, supporting the concept that, in some patients, rising or persistently high Lp(a) may contribute to residual risk and may confound interpretation of achieved “LDL-C” during follow-up. Measurement of Lp(a) concentration in patients resistant to drug hypolipidemic therapy is one of the important modern recommendations concerning Lp(a) determination. In line with contemporary prevention strategies, major European guidelines recommend measuring Lp(a) at least once in each adult person’s lifetime to identify individuals with very high inherited Lp(a) who may carry a lifetime ASCVD risk comparable to heterozygous familial hypercholesterolemia. Such guidance supports the practical feasibility of integrating Lp(a) into routine cardiovascular risk assessment and provides a rationale for interpreting LDL-C results in the context of Lp(a), particularly when LDL-C targets are stringent and clinical decisions are threshold-based. Currently, most international and national clinical guidelines suggest the need to measure Lp(a) concentration at least once to assess vital risk [28]. The world’s first global summit on Lp(a) held in Brussels in March calls for advocating not only the determination of Lp(a) concentration in every individual, but also to perform it at an early age, with full reimbursement from the healthcare system [29]. The assessment of the contribution of Lp(a)-C to the LDL-C determined by a direct method or by calculation, the possibility of considering the adjusted LDL-C both in clinical work and in scientific research, in our opinion, is one of the reasons for including Lp(a) in the lipid panel. Just as the existing limitations of the Friedewald formula are not an obstacle to its long-term use, the use of the LDL-C_corr_, considering Lp(a)-C, can be justified for assessing LDL-C in patients with an increased concentration of Lp(a) at the current stage of development of clinical science. Such an approach will correspond to the principles of personalized medicine and will allow for the determination of individual goals for the prevention and treatment of cardiovascular diseases. The final decision on the need to use the Lp(a) concentration to correct LDL-C requires further discussions by the expert community and large studies that consider all possible limitations of the existing methods for measuring the concentration of Lp(a) and LDL-C.

### Study Limitation

This study has several limitations that should be considered when interpreting the results. First, the analysis was performed on a real-world clinical dataset with repeated measurements: lipid profiles were obtained in patients before and after initiation of lipid-lowering therapy, yielding 566 measurements from 300 patients. Therefore, the observations are not fully independent, and within-subject correlation may have influenced standard errors and agreement metrics.

Second, LDL-C correction for Lp(a) was performed using a fixed conversion factor (Lp(a)-cholesterol estimated as 0.3 × Lp(a), with Lp(a) in mg/dL). However, the cholesterol content of Lp(a) can vary substantially between individuals depending on apo(a) isoform size and particle composition.

Third, this study did not evaluate clinical outcomes. Thus, this work should be interpreted primarily as a laboratory/clinical interpretation study demonstrating that Lp(a)-related correction can materially shift LDL-C category assignment near decision thresholds.

Fourth, because LDL-C was not assessed using a reference method (e.g., β-quantification or a comparable one), we could not determine the true analytical accuracy of the evaluated approaches, and the findings should be interpreted mainly as comparisons of agreement between calculated values.

Fifth, this was a single-center study with a modest sample size, which may limit the generalizability of our results to other settings and populations.

Sixth, the ROC-derived Lp(a) thresholds were developed within this cohort and therefore require external validation before being used for broad clinical decision-making. In addition, because measurements with triglycerides > 4 mmol/L were excluded due to known limitations of the Friedewald equation, the applicability of our findings to patients with marked hypertriglyceridemia may be limited.

## 5. Conclusions

(1) The Friedewald, Martin–Hopkins, and Sampson equations demonstrate high concordance (>95%) for LDL-C calculation in patients with triglyceride levels < 4.0 mmol/L, with 10–13% of values reclassified to higher risk categories when using the Martin–Hopkins and Sampson formulas compared to Friedewald.
(2) The contribution of Lp(a)-cholesterol to calculated LDL-C can reach 90% or more in patients with elevated Lp(a) and low LDL-C levels, resulting in significant systematic errors in LDL-C determination.
(3) Formula-specific threshold Lp(a) concentrations for meaningful LDL-C reclassification were identified:
  - Friedewald formula: Lp(a) ≥ 49 mg/dL (sensitivity, 65%; specificity, 86%; systematic error, 0.81 mmol/L)
  - Martin–Hopkins formula: Lp(a) ≥ 30 mg/dL (sensitivity, 79%; specificity, 73%; systematic error, 0.65 mmol/L)
  - Sampson formula: Lp(a) ≥ 41 mg/dL (sensitivity, 77%; specificity, 80%; systematic error, 0.71 mmol/L)
(4) LDL-C_corr_ should be used in clinical practice for patients with Lp(a) concentrations above the identified thresholds, resulting in reclassification to a lower cutoff level of LDL-C.
(5) These threshold values may support clinical decision-making in patients with increased Lp(a) by indicating when consideration of lipid-lowering therapy intensification is appropriate.

## Figures and Tables

**Table 1:** Characteristics of patients.

Variable	CHD (*n* = 200)	without CHD (*n* = 100)
Male sex, *n* (%)	166 (83%)	62 (62%)
Age at study, years (mean ± SD)	59 ± 9	64 ± 10

**Table 2:** Reclassification of LDL-C values using Martin–Hopkins and Sampson equations compared to Friedewald formula.

	Cut-Off Level of LDL-C (mmol/L)	Number of Results in a Group Relative to Threshold Levels	Number of Reclassified Results According to the Formula (%)	
		Friedewald	Martin–Hopkins	Sampson
1	<1.4	25	10 (40)	9 (36)
2	1.4–<1.8	48	17 (35)	10 (21)
3	1.8–<2.6	129	19 (15)	9 (7)
4	2.6–<3.0	81	10 (12)	8 (10)
5	3.0–<4.0	156	9 (6)	9 (6)
6	4.0–<4.9	63	7 (11)	7 (11)
7	4.9–<6.5	47	4 (9)	4 (9)
8	≥6.5	17	0 (0)	0 (0)
	Over the entire range	566	76 (13%)	56 (10%)

**Table 3:** Number of LDL-C results downgraded using Lp(a)-C adjustment (“LDLCorr”).

Level (mmol/L)	Friedewald			Martin–Hopkins			Sampson		
	LDL-C		RC *	LDL-C		RC	LDL-C		RC
	Used	Corr		Used	Corr		Used	Corr	
<1.4	25	76	0	19	64	0	20	66	0
1.4–<1.8	48	61	21 (44%)	42	62	20 (48%)	49	66	21 (43%)
1.8–<2.6	129	127	49 (38%)	132	127	50 (38%)	129	118	51 (40%)
2.6–<3.0	81	82	44 (54%)	87	81	42 (48%)	82	77	35 (43%)
3.0–<4.0	156	132	60 (38%)	152	140	51 (34%)	151	137	45 (30%)
4.0–<4.9	63	48	31 (49%)	64	48	35 (54%)	64	53	26 (40%)
4.9–<6.5	47	33	24 (52%)	48	34	25 (52%)	49	39	21 (43%)
≥6.5	17	7	10 (59%)	22	10	12 (54%)	22	10	12 (54%)
Total:	566	566	239 (42%)	566	566	235 (42%)	566	566	211 (37%)

* RC—reclassification, presented as *n* (%).

**Table 4:** Agreement between LDL-C category (original vs. corrected) across formulas (8 categories).

	Cohen’s κ	Weighted κ (Quadratic)	Gwet AC1	Po (Observed Agreement)
(a) Friedewald (Lp(a) ≥ 49)	0.046	0.753	0.054	0.169
(b) Martin–Hopkins (Lp(a) ≥ 30)	0.161	0.789	0.185	0.284
(c) Sampson (Lp(a) ≥ 41)	0.121	0.796	0.137	0.242

## Data Availability

The data presented in this study are available upon request from the corresponding author.

## Supplementary Materials

The following supporting information can be downloaded at https://www.mdpi.com/article/10.3390/diseases14020041/s1. Table S1. Reclassification matrix for LDL-C categories: Friedewald (Lp(a) ≥49 mg/dL); Table S2. Reclassification matrix for LDL-C categories: Martin–Hopkins (Lp(a) ≥30 mg/dL); Table S3. Reclassification matrix for LDL-C categories: Sampson (Lp(a) ≥41 mg/dL); Figure S1. Comparison of agreement coefficients for categorical LDL-C versus Lp(a)-corrected LDL-C (LDL-C_corr_) in patients with elevated Lp(a).

## Abbreviations

The following abbreviations are used in this manuscript:

HDL-C	High-density lipoprotein cholesterol
LDL	Low-density lipoprotein
LDL-C	Low-density lipoprotein cholesterol
LDL-C_corr_	Low-density lipoprotein cholesterol corrected to Lp(a) concentration
Lp(a)	Lipoprotein(a)
Lp(a)-C	Cholesterol in Lipoprotein(a)
TC	Total cholesterol
TG	Triglyceride
VLDL	Very low density lipoprotein
