# Supplementary material for: Lipoprotein(a) Concentration and Achieving Target Values of Low-Density Lipoprotein Cholesterol Calculated by Different Equations
Source: Diseases. 2026 Jan 27;14(2):41. doi: 10.3390/diseases14020041 (PMC12939212; doi:10.3390/diseases14020041)
Supplement: Supplementary file 1 [file diseases-14-00041-s001.zip › diseases-4041560-supplementary.pdf]

Table S1. Reclassification matrix for LDL-C categories: Friedewald (Lp(a) ≥49 mg/dL)

A) Cell counts (n).

LDL-C category (mmol/L)| LDL-C_{corr} category (mmol/L)	<1.4	1.4–<1.8	1.8–<2.6	2.6–<3.0	3.0–<4.0	4.0–<4.9	4.9–<6.5	≥6.5
<1.4	6	0	0	0	0	0	0	0
1.4–<1.8	13	1	0	0	0	0	0	0
1.8–<2.6	24	13	5	0	0	0	0	0
2.6–<3.0	5	4	14	0	0	0	0	0
3.0–<4.0	1	5	12	18	8	0	0	0
4.0–<4.9	0	0	0	1	21	3	0	0
4.9–<6.5	0	0	0	2	6	11	5	0
≥6.5	0	0	0	0	0	0	7	4

B) Row percentages (row%).

LDL-C category (mmol/L)| LDL-C_{corr} category (mmol/L) (mmol/L)	<1.4	1.4–<1.8	1.8–<2.6	2.6–<3.0	3.0–<4.0	4.0–<4.9	4.9–<6.5	≥6.5
<1.4	100.0	0.0	0.0	0.0	0.0	0.0	0.0	0.0
1.4–<1.8	92.9	7.1	0.0	0.0	0.0	0.0	0.0	0.0
1.8–<2.6	57.1	31.0	11.9	0.0	0.0	0.0	0.0	0.0
2.6–<3.0	21.7	17.4	60.9	0.0	0.0	0.0	0.0	0.0
3.0–<4.0	2.3	11.4	27.3	40.9	18.2	0.0	0.0	0.0
4.0–<4.9	0.0	0.0	0.0	4.0	84.0	12.0	0.0	0.0
4.9–<6.5	0.0	0.0	0.0	8.3	25.0	45.8	20.8	0.0
≥6.5	0.0	0.0	0.0	0.0	0.0	0.0	63.6	36.4

Notes: LDL-C categories (mmol/L): <1.4; 1.4–<1.8; 1.8–<2.6; 2.6–<3.0; 3.0–<4.0; 4.0–<4.9; 4.9–<6.5; ≥6.5. Rows indicate the uncorrected LDL-C category and columns indicate the corrected LDL-C category. Row% are calculated within each uncorrected LDL-C row (row totals ≈100%). The analysis includes only participants with Lp(a) at or above the method-specific threshold. N=189; unchanged category=32; reclassified=157 (downward shift=157, upward shift=0).

Table S2. Reclassification matrix for LDL-C categories: Martin–Hopkins (Lp(a) ≥30 mg/dL)

A) Cell counts (n).

LDL-C category (mmol/L)| $LDL\text{-}C_{corr}$ category (mmol/L)	<1.4	1.4–<1.8	1.8–<2.6	2.6–<3.0	3.0–<4.0	4.0–<4.9	4.9–<6.5	≥6.5
<1.4	7	0	0	0	0	0	0	0
1.4–<1.8	16	0	0	0	0	0	0	0
1.8–<2.6	20	20	18	0	0	0	0	0
2.6–<3.0	4	6	22	2	0	0	0	0
3.0–<4.0	0	4	12	24	26	0	0	0
4.0–<4.9	1	0	0	1	25	6	0	0
4.9–<6.5	0	0	1	0	6	14	10	0
≥6.5	0	0	0	0	0	1	10	5

B) Row percentages (row%).

LDL-C category (mmol/L)| $LDL\text{-}C_{corr}$ category (mmol/L)	<1.4	1.4–<1.8	1.8–<2.6	2.6–<3.0	3.0–<4.0	4.0–<4.9	4.9–<6.5	≥6.5
<1.4	100.0	0.0	0.0	0.0	0.0	0.0	0.0	0.0
1.4–<1.8	100.0	0.0	0.0	0.0	0.0	0.0	0.0	0.0
1.8–<2.6	34.5	34.5	31.0	0.0	0.0	0.0	0.0	0.0
2.6–<3.0	11.8	17.6	64.7	5.9	0.0	0.0	0.0	0.0
3.0–<4.0	0.0	6.1	18.2	36.4	39.4	0.0	0.0	0.0
4.0–<4.9	3.0	0.0	0.0	3.0	75.8	18.2	0.0	0.0
4.9–<6.5	0.0	0.0	3.2	0.0	19.4	45.2	32.3	0.0
≥6.5	0.0	0.0	0.0	0.0	0.0	6.2	62.5	31.2

N=261; unchanged category=74; reclassified=187 (downward shift=187, upward shift=0).

Table S3. Reclassification matrix for LDL-C categories: Sampson (Lp(a) ≥41 mg/dL)

A) Cell counts (n).

LDL-C category (mmol/L)| LDL-C_{corr} category (mmol/L)	<1.4	1.4–<1.8	1.8–<2.6	2.6–<3.0	3.0–<4.0	4.0–<4.9	4.9–<6.5	≥6.5
<1.4	6	0	0	0	0	0	0	0
1.4–<1.8	17	0	0	0	0	0	0	0
1.8–<2.6	20	18	13	0	0	0	0	0
2.6–<3.0	4	5	17	0	0	0	0	0
3.0–<4.0	0	2	14	20	17	0	0	0
4.0–<4.9	1	0	0	0	19	5	0	0
4.9–<6.5	0	0	0	1	6	14	8	0
≥6.5	0	0	0	0	0	1	10	5

B) Row percentages (row%).

LDL-C category (mmol/L)| LDL-C_{corr} category (mmol/L)	<1.4	1.4–<1.8	1.8–<2.6	2.6–<3.0	3.0–<4.0	4.0–<4.9	4.9–<6.5	≥6.5
<1.4	100.0	0.0	0.0	0.0	0.0	0.0	0.0	0.0
1.4–<1.8	100.0	0.0	0.0	0.0	0.0	0.0	0.0	0.0
1.8–<2.6	39.2	35.3	25.5	0.0	0.0	0.0	0.0	0.0
2.6–<3.0	15.4	19.2	65.4	0.0	0.0	0.0	0.0	0.0
3.0–<4.0	0.0	3.8	26.4	37.7	32.1	0.0	0.0	0.0
4.0–<4.9	4.0	0.0	0.0	0.0	76.0	20.0	0.0	0.0
4.9–<6.5	0.0	0.0	0.0	3.4	20.7	48.3	27.6	0.0
≥6.5	0.0	0.0	0.0	0.0	0.0	6.2	62.5	31.2

N=223; unchanged category=54; reclassified=169 (downward shift=169, upward shift=0).

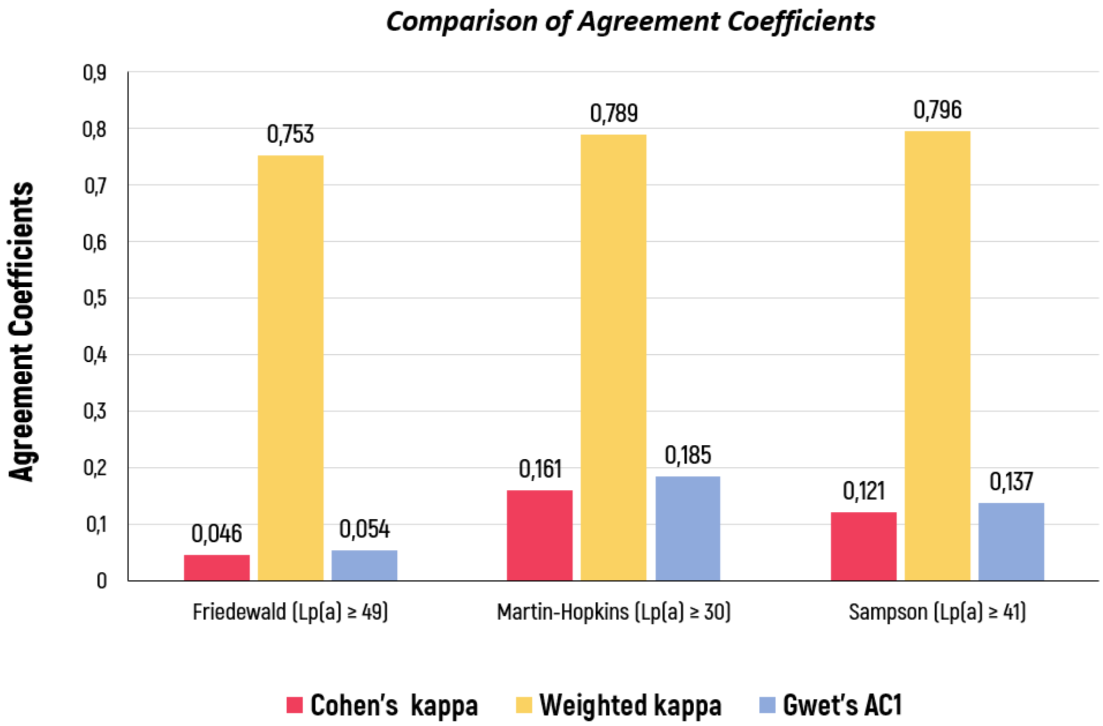

Figure S1. Comparison of agreement coefficients for categorical LDL-C versus Lp(a)-corrected LDL-C (LDL-C_{corr}) in patients with elevated Lp(a). The bar plot summarizes Cohen's kappa (κ), Weighted κ (quadratic), and Gwet's AC1 for three LDL-C equations (Friedewald, Martin-Hopkins, and Sampson) within subgroups defined by Lp(a) concentrations above the ROC-derived cutoffs at which correction for Lp(a)-cholesterol is most likely to change LDL-C category assignment (Friedewald: ≥49 mg/dL; Martin–Hopkins: ≥30 mg/dL; Sampson: ≥41 mg/dL). The consistently low κ and AC1 values indicate poor agreement between LDL-C and LDL-C_{corr} categories at high Lp(a), implying frequent category reclassification after correction and, therefore, potential impact on threshold-based clinical decisions.
